# The Influence of Diet and Its Components on the Development and Prevention of Hepatocellular Carcinoma (HCC)

**DOI:** 10.3390/cancers16051030

**Published:** 2024-03-02

**Authors:** Barbara Janota, Barbara Szymanek

**Affiliations:** 1 Department of Basic Medical Sciences, Faculty of Public Health in Bytom, Medical University of Silesia in Katowice, 41-902 Bytom, Poland; 2 ID Clinic, 41-400 Mysłowice, Poland

**Keywords:** hepatocellular carcinoma, diet, lifestyle, nutrients

## Abstract

**Simple Summary:**

Due to the increasing incidence of hepatocellular carcinoma, which is a global problem, it is valuable to present the latest available literature that describes dietary options for preventing the development of hepatocellular carcinoma and the possibilities of dietary therapy for oncological patients. The authors firstly focus on discussing Metabolic Dysfunction-Associated Steatotic Liver Disease as a factor induced by improper dietary habits, while simultaneously suggesting the adoption of a Mediterranean diet as a model to counteract metabolic disorders resulting in HCC development. Subsequently, the authors discuss the potential for dietary modulation of autophagy processes, the necessity of supporting microbiota, the importance of providing selected vitamins and minerals, as well as compounds particularly valued for their anti-cancer properties, also observed in the case of HCC. The last aspect addressed is the consumption of products contributing to anti-tumor activity.

**Abstract:**

Hepatocellular carcinoma (HCC) is diagnosed annually in nearly a million people worldwide, with approximately half of them being diagnosed at an advanced stage of the disease. Non-infectious risk factors for the development of HCC include an unbalanced lifestyle, including poor dietary choices characterized by a low intake of antioxidants, such as vitamins E and C, selenium, and polyphenols, as well as an excessive consumption of energy and harmful substances. Repeated bad dietary choices that contribute to an unbalanced lifestyle lead to the accumulation of fatty substances in the liver and to it entering an inflammatory state, which, without intervention, results in cirrhosis, the main cause of HCC. This review of the English language literature aims to present the food components that, when included in the daily diet, reduce the risk of developing HCC, as well as identifying foods that may have a carcinogenic effect on liver cells.

## 1. Introduction

Hepatocellular carcinoma (HCC) is a neoplasm whose development, beyond microbiological factors (HBV, HCV infections), depends on lifestyle choices, including dietary choices [1]. HCC develops as a consequence of chronic liver diseases characterized by inflammation and cirrhosis [2]. It is estimated that this type of cancer constitutes over 80% of diagnosed primary liver tumors. The frequency of diagnosis is on the rise, concurrently increasing the need for diagnostics among individuals with elevated risk, aiming for early detection [3]. Considering the significant impact of daily lifestyle-related factors on disease development, there seems to be a necessity to explore preventive measures that, when implemented in daily life, could contribute to a reduction in incidence. Among these measures, particular attention should be directed towards daily dietary behaviors influencing metabolic health and, through the bioactivity of nutritional components, gene expression, as extensively described through nutrigenomics [4,5].

The development of cancer as a result of chronic metabolic abnormalities is attributed to recurring poor dietary choices, leading to excessive body weight, predisposing the individual to the accumulation of fat compounds in liver cells [1]. The accumulation of fat droplets in hepatocytes results in the development of non-alcoholic fatty liver disease [1]. Its progression may be accompanied by inflammation (non-alcoholic steatohepatitis), currently considered a major cause of mortality due to hepatocellular carcinoma [6].

The non-metabolic causes of liver cell carcinogenesis relate to the intake of substances with mutagenic effects through diet, adversely affecting gene expression, and to engagement in a diet characterized by insufficient antioxidant potential [2,7,8]. As a result of these abnormalities, a diet lacking essential substances for detoxification and repair processes may promote the development of the cancer process [2,7,8].

Because of the increasing incidence of HCC and the observed connections between nutrition and liver cell carcinogenesis, there is a need to systematize information regarding the pro- and anti-carcinogenic potential of food and dietary practices in order to recognize threats and implement preventive actions. This literature review aims to present the impact of diet and its components on preventing and developing hepatocellular carcinoma. The manuscript provides evidence of the potential impact of certain natural food products on the development and prevention of HCC. Based on the presented publications, it is possible to plan further research into characterized products in the context of HCC, as well as implementing recommendations into patients’ daily routines.

## 2. Materials and Methods

The literature review was performed in December 2023. Articles were searched in the PubMed database and Google Scholar database, using the phrase “hepatocellular carcinoma” and “nutrients”. The review included 108 publications, of which 102 were published in 2019–2024 and 6 were published in 2010–2018. The last search was performed on 30 December 2023.

## 3. Nutrition-Dependent Metabolic Dysfunction-Associated Steatotic Liver Disease (MASLD) as a Risk Factor for HCC

The global prevalence of MASLD is estimated to be 32% [9]. This percentage is steadily growing, and its nutritional basis is attributed to an excessive energy intake, characteristic of the Western dietary pattern [10]. The accumulation of fat compounds in the liver is promote by irregular meal consumption, an excessive intake of monocarbohydrates and saturated fats, and a simultaneous low intake of dietary fiber [11,12]. These errors, accompanying the development of visceral obesity and dyslipidemia, result in insulin resistance and the stimulation of de novo lipogenesis in the liver [12,13]. The primary aim of prevention should be to prevent unplanned weight gain and the development of lipid disorders [14]. As reported by Saitta et al., merely having excess body weight increases the risk of HCC by at least several percent [15]. Hence, the daily diet should be normocaloric and provide nutrients in accordance with individual requirements [14]. In the case of the development of the described disorders, a worsening in the lipid parameters of body composition, and an increase in waist circumference, the implementation of a low-calorie diet is necessary to ensure weight reduction [16]. The Mediterranean diet (MD) is recommended as a dietary model in case of the need to improve liver parameters, the deterioration of which is observed among overweight individuals. Its adoption contributes to the reduction of inflammation, an improvement to liver stiffness parameters, a reduction in total cholesterol levels, and also a reduction in waist circumference [17,18]. The MD relies on the intake of primarily monounsaturated fatty acids through the use of olive oil, as well as the intake of polyunsaturated fatty acids through the consumption of fatty fish [17]. These fatty acids are characterized by the suppression of pro-inflammatory cytokines, which are observed in MASLD. Other characteristic components of the MD include soluble fiber and bioactive compounds provided by vegetables and plant products [17]. It has been proven that a higher intake of dietary fiber is associated with a reduced risk of MASLD, while some of the bioactive compounds, by influencing metabolic processes, decrease de novo lipogenesis and enhance lipolysis, counteracting the development of disorders that gradually lead to HCC [17].

A diet to prevent the development of MASLD should regularly include vegetables, fruits, legumes, fiber-rich products, and sources of unsaturated fats [17]. Conversely, products rich in saturated fats, low-fiber products providing refined carbohydrates, and those with added fructose should be eliminated from the diet [17]. Therefore, to minimize the risk of HCC development, efforts should be directed towards maintaining proper body composition parameters and basic test results, such as in lipid profile and liver function tests, through adherence to the general principles of healthy nutrition [18,19].

## 4. Potential of Fasting-Activated Autophagy

An alternative method for cancer prevention involves harnessing the natural repair mechanisms occurring within cells, activated through diet. An example of such a mechanism is autophagy, a process that eliminates faulty proteins and organelles, while also targeting energy components to ensure metabolic and cellular homeostasis [20]. Disorders in autophagy can occur due to mutations in the ATG5 and ATG7 genes, as well as excessive expression of HIF1A-AS1 [21]. Impaired autophagy function in lysosomes is observed in MASLD, and in the development of HCC, autophagy operates inefficiently, leading to ineffective tumor suppression [20]. The possibility of regulating autophagy processes in the liver has become the subject of numerous studies aiming to explore ways to intensify it, ultimately influencing cancer inhibition. It has been demonstrated that autophagy processes are intensified, among other situations, during stress induced by nutrient deficiencies [22].

Mimicking a stressful situation through periodic nutrient deprivation occurs during intermittent fasting practiced in various ways. One common approach to intermittent fasting involves intentionally withholding nutrients at specific times of the day [22]. Other patterns may include nutrient deprivation for one day a week or for several days, with unrestricted food intake during non-fasting periods [22]. It has been observed that intermittent fasting positively influences lipid parameters and carbohydrate metabolism, reduces inflammatory processes, reduces leptin resistance, and may also support oncologic therapy in HCC [22,23].

Studies on the effectiveness of sorafenib in HCC treatment prompted researchers to evaluate intermittent fasting in cells, organoids, and mice with human-induced HCC (heterotransplantation), anticipating an intensified therapeutic effect [24]. Ultimately, the sensitization of cancer cells to the action of the drug was observed [24].

## 5. Malnutrition as a Condition Requiring Intervention in the Prevention and Treatment of HCC

Hepatocellular carcinoma (HCC) develops as the final stage of organ damage, preceded by chronic inflammation leading to cirrhosis. Liver cirrhosis necessitates careful dietary management due to numerous gastric–metabolic disorders and improper diet balance, which may lead to life-threatening malnutrition and consequent sarcopenia [25,26]. Factors conducive to malnutrition include reduced hunger sensation due to elevated leptin levels, ascites, inflammatory conditions, increased catabolism, and the need for gluconeogenesis, also utilizing branched-chain amino acids (BCAAs), namely, isoleucine, leucine, and valine, typically used for muscle tissue building [25]. Notably, nutrients impact immune functions and immunotherapy in HCC [27]. As highlighted in the review by Zhang C et al., asparagine deficiency disrupts the functioning of CD8 lymphocytes, while its excess enhances their anti-tumor activity [27]. Inhibiting glutamine breakdown likely activates this subset of lymphocytes, as well [27]. Serine is essential for T lymphocyte proliferation, while creatine contributes to slowing down the proliferation of cancer cells, similar to tryptophan, which enhances the cytotoxicity of the immune system cells [28]. It has also been demonstrated that glutamine deficiency contributes to the impairment of CD8 lymphocyte function in hepatocellular carcinoma [29]. Researchers emphasize the need for rapid nutritional diagnostics and the implementation of nutritional therapy, including an optimal protein intake ranging from 1.2 to 1.5 g/kg per day among HCC-diagnosed patients due to the risk of malnutrition and sarcopenia development, adversely affecting the tolerance of surgical treatment [30,31,32]. High-protein foods, such as meat, dairy, and eggs, serving as a source of complete and highly bioavailable protein containing all essential amino acids, should be considered. Additionally, supplementation with BCAA-containing preparations, which raise serum albumin levels and positively impact the quality of life in HCC patients, should also be considered [33].

## 6. Nutritional Support for the Microbiota

In recent years, there has been growing interest in the associations between the microbiota and pathogenicity, as well as its supportive role in health. The microbiota comprises bacteria, fungi, archaea, and protozoa [34]. Current research focuses on evaluating the impact of bacteria and their harmful metabolites in the intestines and hepatocytes. This influence is possible through the portal circulation, where, due to a disrupted intestinal barrier, bacteria can enter the liver [35,36]. Harmful metabolites include butyrate, long-chain fatty acids, endotoxins, and indole-3-octanoic acid [37]. However, a clear link between dysbiosis and hepatocellular carcinogenesis has not yet been identified [36]. Among patients who reached HCC as the final stage of liver cirrhosis, an increase in the abundance of *Bacteroides*, *Ruminococcus*, and *Enterobacteriaceae* in the intestines was observed, accompanied by a decrease in *Bifidobacterium* and *Akkermansia* abundance, both of which negatively correlated with calprotectin levels, a marker of intestinal inflammation [33,38]. Dysbiosis is also associated with a decrease in serum phosphatidylcholine concentration, favoring the accumulation of fat compounds in the liver [33].

As a result of these observations, the possibility of providing probiotic bacteria and prebiotic substances to prevent pathogenic processes, including those leading to HCC, is being explored [36]. Probiotic bacteria reducing the risk of HCC development have been described by Wass Thilakarathna WP.D. et al. in their review [39]. Among the mentioned strains are *Lactobacillus* spp. and *Bifidobacterium* spp., which exhibit protective functions by reducing the expression of pro-inflammatory molecules such as TNF-alpha, improving histological images, and generally lowering inflammatory markers [39]. These strains are commonly used to restore homeostasis and strengthen the intestinal barrier through both supplementation and dietary means. Fermented vegetables, fruits, and dairy products, including yogurts and kefirs, are rich in *Lactobacillus* spp. [40,41]. In addition to strengthening the intestinal barrier, probiotic strains found in yogurt contribute to binding toxic substances, including aflatoxins, resulting in a reduced absorption of potentially carcinogenic substances, and they also decrease the absorption of harmful lipopolysaccharides, thereby reducing the risk of organ inflammation [42,43,44]. Probiotics provided through fermented plant-based foods may also act at the foundation of metabolic disorders, improving the carbohydrate and lipid metabolism, as well as body composition [45]. Thus, the consumption of fermented plant-based products and dairy may have protective effects against HCC development and enhance the therapeutic process.

## 7. Vitamins and Trace Elements as Supportive Elements in the Prevention of, and Therapy for, HCC

The supply of vitamins and trace elements through diet is essential for various physiological processes, with some exerting specific influences on liver function. Deficiencies in these nutrients can disrupt the metabolic and detoxification functions of the organ, leading to chronic diseases [46]. To prevent a deficiency in essential components, supplementation is necessary in certain cases [47,48].

### 7.1. Fat-Soluble Vitamins

Vitamin D3 is a pleiotropic vitamin with diverse properties. Aside from its impact on calcium–phosphorus metabolism and bone mineralization, Grinberg L. lists its anti-inflammatory, immune-system-regulating, fibrosis-inhibiting, and lipid-metabolism-regulating actions. Additionally, studies on animal models have demonstrated its ability to limit fat accumulation in the liver, which is noteworthy in preventing further consequences of the disease [49]. There are also reports of its anti-cancer properties [50]. The primary natural source of vitamin D3 for humans is exposure to sunlight, while dietary sources include egg yolks, fish, seafood, dairy, and vitamin-D3-fortified products [51]. Dietary intake and sunlight exposure often prove insufficient, meaning that vitamin D3 supplementation is recommended to achieve blood levels ranging from ≥20 ng/mL to 100 ng/mL [52].

Due to the broad actions of vitamin D3 and its liver activation, scientists have assessed its potential impact on HCC development. In a study on a mouse model, Matsuda A. et al. did not observe therapeutic effects on the discussed type of cancer [53]. However, Yonggui Zhang observed, in a meta-analysis, that an increase in serum D3 levels is associated with a reduced risk of HCC development [54]. Immunomodulatory effects from vitamin D3 supplementation have also been observed among individuals with HCC, affecting cytokine and chemokine production. This recognition positions vitamin D3 as a supportive element in cancer treatment [55]. The immunomodulatory and anti-fatty-liver effects should encourage both physicians and patients to strive for normal vitamin D3 concentrations in the blood, to potentially exert a preventive influence on HCC.

Another vitamin considered in the context of HCC treatment is vitamin K2 (menaquinone), sourced from dairy, eggs, and meat [56]. It is also produced in the human body with the involvement of probiotic bacteria, emphasizing the importance of maintaining a healthy microbiota [56,57]. The daily requirement for vitamin K in Central European countries is 65 µg for men and 55 µg for women [57]. Studies conducted on individuals treated with sorafenib, along with the oral administration of vitamin K2 at a dose of 45 mg, showed enhanced anti-cancer activity in the medicine, supporting the benefits of including K2 in the diet [58].

When discussing fat-soluble vitamins, we should also mention vitamins A and E, with the latter comprising tocopherols and tocotrienols. Dietary sources of vitamin E include nuts, almonds, plant oils (especially olive oil), and sprouts [59]. Although there is no available research indicating the anti-cancer effects of vitamin E on HCC development or progression, its anti-inflammatory properties are utilized in the treatment of liver steatosis and inflammation. It also enhances insulin sensitivity, suggesting a protective role against the metabolic consequences of liver disorders [60].

Researchers evaluating the impact of α-tocopherol and β-carotene supplementation as a provitamin A on the incidence and mortality of HCC did not demonstrate a reduced risk of the disease. However, synthetic retinoids, derivatives of vitamin A, are considered a therapeutic possibility in HCC, and their deficiency contributes to inflammation and organ steatosis. Dietary sources of these retinoids include carrots, pumpkins, parsley, and peaches [57].

### 7.2. Coenzyme Q10

Coenzyme Q10 (CoQ10), participating in the electron transport, is necessary in energy production [61]. This compound is naturally produced by the body up to around the age of 20, after which its synthesis decreases. CoQ10 can be obtained from the diet by consuming meat, liver, eggs, fish, and egg yolks [62]. It exhibits antioxidant properties, inhibiting the generation of free radicals by enhancing the activity of superoxide dismutase, catalase, and glutathione peroxidase. As a result, it is considered a compound with anti-cancer properties, including the inhibition of the proliferation of HCC cells [62,63].

### 7.3. Trace Elements

Zinc and selenium are trace elements with specific immunomodulatory properties. Zinc is found in grains, legume seeds, eggs, meat, and dairy products [64]. It is involved in various biochemical processes, contributing to cell division, immune responses, and antioxidative reactions [65,66]. Excess amounts of zinc are stored in the liver, where they facilitate the functioning of the ornithine cycle, including the activation of glutaminase dehydrogenase [65,66]. Zinc levels in the blood are reduced among individuals suffering from liver diseases [66]. Clinical studies have observed the beneficial impact of zinc supplementation among people with chronic liver diseases, reducing the risk of HCC occurrence and improving organ function [66]. Furthermore, it is suggested that the calculation of the copper-to-zinc ratio may be used to estimate the survival of HCC patients, as zinc levels decrease with disease progression, and among individuals with a better prognosis, the ratio is ≥0.999 [64].

Selenium is part of numerous enzymes, including being a cofactor for glutathione peroxidase, an antioxidative enzyme. Its concentration is considered a factor influencing the frequency of HCC recurrences [67,68]. Metabolically, selenium deficiency results in increased fat accumulation in the liver and intensified insulin resistance [69]. Research on soil selenium saturation indicates deficiencies in a significant percentage of the study material, corresponding to selenium deficiencies in food [70,71]. These deficiencies reflect low selenium saturation in living organisms and are associated with various health consequences, including an increased risk of cancer [72]. Dietary sources of selenium include grain products, nuts, meat, eggs, and dairy products, with the element’s concentration depending on the animal’s diet [72]. Scientists indicate that selenium exhibits anti-inflammatory effects and inhibits the proliferation of cancer cells in the case of HCC. It directs them towards apoptotic pathways, making it a supportive microelement in therapy [73]. Dietary supplementation to address selenium deficiencies appears to be the most favorable method due to its toxic effects resulting from excessively high doses [74]. Achieving the optimal human body saturation with selenium is also considered a factor in reducing the risk of HCC development, as individuals with the disease have been observed to have lower selenium levels compared to healthy individuals [74].

## 8. Plant Hepatoprotective Compounds

Polyphenols are compounds found in plant products that primarily exert their effects through antioxidation, the antiproliferation of altered cells, and protection against mutations [75]. By influencing intracellular signals, polyphenols demonstrate anti-angiogenic properties, thereby reducing the risk of metastasis in HCC [75]. The impact of polyphenols on HCC development is also attributed to the initiation of apoptosis in cancerous cells. Kwok-Chui Cheng et al. assessed the influence of polyphenols from white mulberry fruits on HCC development, concluding that the extract from white mulberry fruits could be used in preventing this cancer [76].

Curcumin, a component of the commonly used spice in Asian cuisine, turmeric, extracted from the rhizome of turmeric, exhibits anti-cancer effects in HCC. Apart from its anti-cancer properties, curcumin also offers anti-inflammatory characteristics [76,77,78]. Its pro-apoptotic effects on cancer cells have been demonstrated, along with its beneficial impact on glucose tolerance and anti-steatosis in the liver. Scientists suggest its use as a functional food ingredient due to its inhibitory effects on the mitotic division of cancer cells [78].

Kardamonin, also present in the popular spice cardamom, is characterized by anti-cancer properties, as demonstrated in breast and ovarian cancer cases [79]. Nassrin A Badroon et al. investigated its in vitro effects on HCC cell proliferation, obtaining results indicating both antiproliferative and pro-apoptotic actions [79]. Researchers concluded that kardamonin could be considered as an alternative form of oncological therapy in HCC [79].

Quercetin is another compound with properties that can be used in cancer prevention, including HCC prevention, by counteracting hepatocyte damage, particularly in cases of exposure to toxic substances [80,81]. The substance mitigates inflammation and fibrosis in the liver [82]. A meta-analysis conducted by Paul Fernández-Palanca et al. indicated that quercetin has antiproliferative and pro-apoptotic effects on HCC cells [83].

Cytotoxic effects on HCC cells have also been observed with berberine [84]. Berberine is an alkaloid naturally occurring in the fruits of the barberry shrub, often consumed in dried form and popularly used as an immune-boosting food. The substance induced apoptosis in the organelles of diseased cells [84]. In an animal model with developed HCC, the application of berberine reduced the proliferation of cancer cells and acted anti-angiogenically, preventing metastasis to the respiratory system [85]. In other studies, it was observed that HCC cell growth was impossible without the involvement of GPT1, one of the isoforms of alanine transaminase responsible for metabolic processes [86]. Utilizing this information, scientists demonstrated that berberine inhibits GPT1, resulting in the inhibition of ATP synthesis in cancer cells [86].

Lycopene is an ingredient in many commonly consumed products, and its cancer prevention properties have been extensively described in the scientific literature [87]. It is a carotenoid found in its highest concentration naturally in tomato skin, but is also present in tomato-based products such as ketchup, strained tomatoes, and passata. Due to its described anti-cancer properties, research is being conducted to assess its impact on HCC. In a meta-analysis of studies conducted on rodents, presented by Abraham Nigussie Mekuria et al., it is inferred that lycopene acts as an anti-cancer agent and hinders disease progression [88]. Among the mechanisms mentioned for this action, authors cite the inhibition of enzymes initiating carcinogenesis processes, a reduction in oxidative stress, and the regulation of gene expression responsible for cell division [88].

Kaempferol, a hepatoprotective polyphenol, is found in its highest concentrations in pumpkin, chives, spinach, kale, and dill [89]. In animal studies, its protective effects in cases of liver damage have been proven, showing anti-fibrotic and inhibitory effects on the accumulation of fatty substances in the organ [89]. In HCC, kaempferol has promoted apoptosis in diseased cells and reduced inflammation [89]. Products rich in this compound should be particularly considered as preventive measures in liver diseases, including HCC.

Epigallocatechin gallate (EGCG), found in green tea leaves, is subjected to research to evaluate its potential use as an anti-cancer substance due to its antioxidative properties [90]. It was established that the polyphenol counteracts liver inflammation [90]. In an animal model study inducing liver cirrhosis, EGCG reduced the values of liver fibrosis risk markers by deactivating stellate cells [90]. Authors inferred that the substance counteracts organ damage and green tea itself is a beverage protecting against HCC development [90,91].

Sulforaphane is found in its highest concentrations in vegetables such as cabbage, cauliflower, and broccoli. Its anti-cancer properties have been repeatedly proven [89]. In addition to its antioxidative and cell-protective effects, its particular property is preventing angiogenesis in the tumor and the entire liver area, preventing fibrosis [82]. This is possible through the inhibition of vascular endothelial growth factor secretion and activation of Nrf2 (nuclear factor erythroid 2-related factor 2), which acts protectively on cells [82].

## 9. Selected Food Products with Hepatoprotective Properties

### 9.1. Coffee

Recommendations regarding coffee consumption often undergo changes as researchers continuously study its properties. The main components attributed to modulating the body’s health are caffeine and numerous polyphenols, with chlorogenic acid being the most frequently described [92]. Chlorogenic acid has the property of eliminating reactive oxygen species [92]. A meta-analysis revealed that consuming >2 cups of coffee per day reduces the risk of HCC by 35% [93]. Similar conclusions were drawn by Yunyang Deng et al., who found that more frequent coffee consumption was associated with a lower risk of HCC [94]. Researchers present this beverage as a potential preventive measure for HCC, similar to green tea [91].

### 9.2. Honey

Valued for its broad health-promoting properties, honey has also been studied for its protective influence on liver cells. As a substance produced by bees, it contains secretions from these insects, as well as flower pollen, making it a unique product with an unparalleled chemical composition [95]. The main immune-stimulating components in honey include proteins, enzymes, and polyphenols [95]. In a study by Hamed A Ghramh et al., the impact of honey and silver-nanoparticle-infused honey on HCC cell growth was beneficial, suggesting that the substance slows down the tumor’s growth processes [95].

### 9.3. Chlorella

Chlorella has become a popular superfood in recent years. It is consumed as an additive in shakes in powder form and used as a dietary supplement [96]. Chlorella is a single-celled algae whose main property is detoxifying tissues from heavy metals and providing nutrients and vitamins [96]. The impact of ethanol and polysaccharide extracts from algae was evaluated by Nunghathai Sawasdee et al. in terms of HCC cell survival, with the results indicating anti-cancer effects [97]. Carotenoids and epigallocatechin gallate have been identified in studies using the applied extract [97]. Ragaa A. Hamouda et al. demonstrated the anti-cancer effect of methanol extract from chlorella on HCC [98]. In animal studies with developed non-alcoholic fatty liver disease, alga supplementation improved the insulin sensitivity and lipid profile and reduced inflammation [99]. This study suggests the use of algae to protect from the exacerbation of metabolic disorders in MASLD.

### 9.4. Interesting Potential of Mushrooms in HCC Prevention and Treatment

Mushrooms are commonly used in the daily cuisine of many countries worldwide. Beyond their culinary use due to their flavor profiles, they are a source of health-promoting substances that support therapies for many lifestyle diseases rooted in metabolic disorders [100]. Among the properties exerted by mushrooms (shiitake) are the improvement of leptin sensitivity, a reduction in hunger, and the inhibition of liver fat deposition [100]. The literature describes reductions in lipoprotein levels and liver steatosis, as well as anti-inflammatory effects in the organ (reishi) [100]. In xenotransplantation models of HCC, reishi extract induced apoptosis pathways and extracts from shiitake and maitake contributed to tumor volume reduction [100]. In studies on reishi, Ming Song et al. proved that the extract inhibits HCC cell proliferation by interacting with immune cells, promoting apoptosis [101].

Single-celled fungi are also the subjects of research. β-D-glucan, a substance found in yeast, is known for its anti-inflammatory, immune-modulating, and anti-cancer properties [102]. Ningning Wang et al. conducted research on the impact of β-D-glucan on autophagy and apoptosis processes in mouse-induced HCC [102]. Researchers observed that the substance present in yeast promotes HCC cell apoptosis [102]. Expanding our knowledge about the beneficial properties of edible mushrooms could contribute to preventing HCC development and supporting therapy, especially in the case of β-D-glucan, for which scientists declare a lack of toxicity and an absence of observed side effects in therapy [102].

The hepatoprotective potential of food is presented in Table 1.

## 10. Foods and Food Compounds of Which Consumption Should Be Avoided

### 10.1. Alcohol

Alcohol, being a highly toxic and carcinogenic substance, is considered a factor contributing to liver inflammation and fibrosis [103]. A literature review indicates the necessity for individuals with non-alcoholic fatty liver disease to abstain from alcohol consumption, because of a proven reduction in HCC risk in this group [104]. Similar conclusions were drawn in a cohort study by Fredrik Åberg et al., presented in their original work [105].

### 10.2. Iron Overload in At-Risk Groups

Controlling iron intake in daily diet among individuals with chronic liver diseases has a significant preventive impact in HCC. Iron is an element stored in the liver and has oxidative properties [106]. Disturbances in its metabolism, occurring among individuals with hemochromatosis and chronic liver diseases, can initiate oncogenic processes leading to HCC [106].

### 10.3. Aflatoxins

Aflatoxins are substances produced by certain fungi of the *Aspergillus* spp. They are mutagenic, carcinogenic substances. Exposure to them is a cause of hepatocellular carcinoma (HCC) [107]. Products at risk of contamination include those exposed to moisture, with grains and nuts being particularly susceptible [107]. Minimizing exposure to aflatoxins may involve choosing local or fresh products that have not undergone prolonged storage, thereby limiting conditions conducive to mold growth [108].

## 11. Conclusions

The literature presented indicates the potential of diet and its components in preventing the development of hepatocellular carcinoma. Among the elements of dietary prevention and dietary therapy, depending on the patient’s condition, the following should be highlighted:

- Implementation of a diet preventing liver steatosis, enabling the attainment of proper body composition parameters, with antioxidant potential;
- Optimal nourishment of the body, with a particular emphasis on an appropriate intake of amino acids;
- Nutritional support for the microbiota through the provision of probiotics and food-based prebiotics;
- Consumption of products rich in fat-soluble vitamins, CoQ10, zinc, and selenium;
- Adoption of a diet rich in diverse vegetables, fruits, and mushrooms, as sources of bioactive compounds;
- Inclusion of coffee, honey, and hepatoprotective superfoods in one’s daily diet;
- Avoiding the consumption of products that may increase the risk of supply substances harmful to the liver.

**Figure 1:**
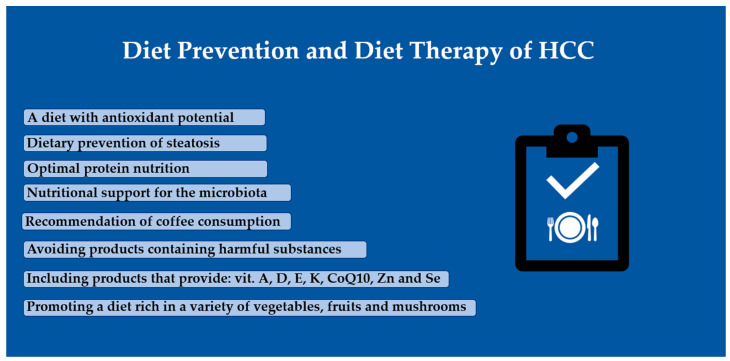
Dietary elements for the prevention of, and therapy for, HCC.

Patients and healthcare workers should be educated about the role and importance of diet; this could be preventively implemented at the stage of diagnosis of chronic liver disease and adapted to its etiology. This applies especially to patients with liver cirrhosis, who have a significantly increased risk of developing liver cancer. The therapeutic strategy in HCC could be expanded to include elements of diet therapy, which may also be helpful in reducing the negative effects of oncological treatments (Figure 1).

The literature review outlines potential dietary therapy paths and preventive measures that can be applied by dietitians and physicians among individuals with an increased risk of HCC.

## Figures and Tables

**Table 1:** Hepatoprotective potential of food.

Type of Food	Components	Functions	References
Fermented vegetables and dairy	*Lactobacillus* spp. and *Bifidobacterium* spp.	- Reduction in pro-inflammatory molecule expression, TNF-alpha. - Improvement of liver histological images. - Decrease in the absorption of potentially carcinogenic substances. - Reduction in the absorption of harmful lipopolysaccharides. - Improvement of carbohydrate and lipid metabolism.	[39,42,43,44,45]
Meat, dairy, eggs	High-protein products	- Increase in serum albumin concentration. - Improvement of surgical treatment tolerance. - Decrease in the risk of immune function disorders. - Lowering the risk of sarcopenia.	[27,28,29,30,31,32,33]
Egg yolks, fish, dairy, seafood	D3	- Therapeutic impact in HCC. - Reduction of HCC risk. - Immunomodulation and anti-lipid effects.	[53,54,55]
Dairy, eggs, meat	K2	- Potentiation of the anti-cancer drug sorafenib.	[58]
Nuts, almonds, plant oils, sprouts, olive oil	Vitamin E	- Anti-inflammatory action. - Improvement of insulin sensitivity.	[59,60]
Meat, liver, egg yolks, fish	CoQ10	- Antioxidation, inhibition of free radical generation: enhances the activity of superoxide dismutase, catalase, and glutathione peroxidase. - Anti-cancer and antiproliferative properties.	[62,63]
Grains, legume seeds, eggs, meat, dairy products	Zinc	- Reduction in HCC occurrence risk and improvement of liver function.	[66]
Whole grains, nuts, meat, eggs, dairy products	Selenium	- Anti-inflammatory action. - Inhibition of cancer cell proliferation and induction of apoptotic pathways in HCC.	[72,73]
White mulberry	Polyphenols	- HCC prevention.	[76]
Turmeric	Curcumin	- Proapoptotic impact in HCC. Improvement of glucose tolerance, reduction in liver steatosis. - Antiproliferative effect.	[78,80]
Cardamom	Cardamonin	- Antiproliferative and proapoptotic effects in HCC.	[79]
Apples, grapes, citrus fruits	Quercetin	- Anti-inflammatory and anti-fibrotic effects. - Antiproliferative and proapoptotic impact on HCC.	[82,83]
Barberry fruits	Berberine	- Antiproliferative, anti-angiogenic, inhibitory impacts on GPT1.	[84,85,86]
Tomatoes and tomato products	Lycopene	- Inhibition of enzymes initiating carcinogenesis processes. - Reduction in oxidative stress. - Regulation of gene expression responsible for cell divisions.	[82]
Pumpkin, chives, spinach, kale, dill	Kaempferol	- Anti-fibrotic effects and inhibition of fatty substance accumulation.	[89]
Green tea leaves	Epigallocatechin gallate	- Anti-inflammatory action, reduction in fibrosis risk markers.	[90]
Cabbage, cauliflower, broccoli	Sulforaphane	- Inhibition of vascular–endothelial growth factor secretion and activation of Nrf2.	[82]
Coffee	Chlorogenic acid	- Reduction in HCC risk.	[93]
Honey	Proteins, enzymes, polyphenols	- Slowdown of tumor growth processes.	[95]
Chlorella	Algae	- Preventive against HCC. - Improvemet of insulin sensitivity, lipid profile. - Reduction in inflammation.	[97,98,99]
Shiitake, reishi, yeast, pine mushroom, king oyster mushroom	Various bioactive compounds, including β-D-glucan	- Improvement of leptin sensitivity, reduction in liver steatosis. - Anti-inflammatory effect. - Inhibition of proliferation, promotion of apoptosis, modulation of immune response.	[100,101,102]
